# Correction: Palmitoylated SCP1 is targeted to the plasma membrane and negatively regulates angiogenesis

**DOI:** 10.7554/eLife.32342

**Published:** 2017-10-17

**Authors:** Peng Liao, Weichao Wang, Yu Li, Rui Wang, Jiali Jin, Weijuan Pang, Yunfei Chen, Mingyue Shen, Xinbo Wang, Dongyang Jiang, Jinjiang Pang, Mingyao Liu, Xia Lin, Xin-Hua Feng, Ping Wang, Xin Ge

Liao P, Wang W, Li Y, Wang R, Jin J, Pang W, Chen Y, Shen M, Wang X, Jiang D, Pang J, Liu M, Lin X, Feng X-H, Wang P, Ge X. 2017. Palmitoylated SCP1 is targeted to the plasma membrane and negatively regulates angiogenesis. *eLife* **6**:e22058. doi: 10.7554/eLife.22058.

Published 31, May 2017

We would like to thank Dr. Caiyun Fang and Haojie Lu, who pointed out the omission of the citation in the original article.

In the published article, the articles shown below should be cited in the Results.

C Fang, X Zhang, L Zhang, X Gao, P Yang, H Lu (2016). Identification of Palmitoylated Transitional Endoplasmic Reticulum ATPase by Proteomic Technique and Pan Antipalmitoylation Antibody. *Journal of Proteome Research* 15(3): 956-962.

X Wei, H Song, CF Semenkovich (2014). Insulin-regulated protein palmitoylation impacts endothelial cell function. *Arterioscler Thromb Vasc Biol.* 34(2): 346-354.

In the fourth paragraph of the subsection “Palmitoylation of SCP” of the Results, the text “the palmitoylation of SCP1 can be regulated by both 2BP and palmostatin B in an opposing fashion, as shown by the decreased palmitoylation of SCP1 by 2BP versus the increased palmitoylation of SCP1 by palmostatin B (Figure 2H)” has been replaced with “the palmitoylation of SCP1 can be regulated by both 2BP and palmostatin B in an opposing fashion, as shown by the decreased palmitoylation of SCP1 by 2BP versus the increased palmitoylation of SCP1 by palmostatin B via using pan Anti-palmitoylation Antibody (Fang C et al., 2016) (Figure 2H)”.

In the subsection “Antibodies, reagents, immunoprecipitation and western blotting” of the Materials and methods, the text “A total of 25 mg of total protein was separated by SDS-PAGE and blotted with anti-β-actin (Santa Cruz 47778, RRID: AB-626632)” has been replaced with “A total of 25 mg of total protein was separated by SDS-PAGE and blotted with Pan Anti-palmitoylation (from Haojie Lu), anti-β-actin (Santa Cruz 47778, RRID: AB-626632)”.

In the Acknowledgements, the text “We thank Dali Li, Meizhen Liu, and other members of the Wang laboratory for their technique assistance.” has been replaced with “We thank Dali Li, Meizhen Liu, and other members of the Wang laboratory for their technique assistance. We thank Dr. Caiyun Fang and Dr. Haojie Lu for providing the Pan Anti-palmitoylation Antibody to us.”

In the fifth paragraph of the Introduction, the text “Moreover, palmitoylation is intimately involved in signaling networks by interacting with various protein molecules such as insulin in the control of phenotypic and functional changes of endothelial cells (Fang et al., 2014)”has been replaced with “Moreover, palmitoylation is intimately involved in signaling networks by interacting with various protein molecules such as insulin in the control of phenotypic and functional changes of endothelial cells (Wei et al., 2014)”.

We apologize for any confusion that may have occurred.

The article has been corrected accordingly.

